# IL-10 Modulates *In Vitro* Multinucleate Giant Cell Formation in Human Tuberculosis

**DOI:** 10.1371/journal.pone.0077680

**Published:** 2013-10-17

**Authors:** Parul Shrivastava, Tamishraha Bagchi

**Affiliations:** Department of Microbiology and Biotechnology Centre, Faculty of Science, the Maharaja Sayajirao University of Baroda, Vadodara, India; National Center for Cell Science, India

## Abstract

**Background:**

Multinucleated giant cells (MGC) are the histologic hallmark of granuloma which is known to limit tuberculosis infection. Both Th1 and Th2 type of cytokines regulate the immune response occurring within the granulomas. The objective of the study was to determine whether tuberculosis patient monocytes differed in their MGC forming ability as compared to healthy controls.

**Methods:**

In vitro MGC formation was carried out by treatment of monocytes with cytokine containing culture supernatant of ConA or PPD stimulated peripheral mononuclear cells. IL-2, TNF-α, IL-4, IL-10 and TGF-β cytokine levels were analysed in culture supernatants using ELISA. IL-4 and IL-10 were added to culture supernatant separately and simultaneously along with their respective neutralizing antibodies and their consequent effect on MGC formation was evaluated.

**Results:**

MGC formation was significantly low in patient monocytes incubated with autologous culture supernatant as compared to control culture supernatant. Cytokine analysis of the culture supernatants revealed that while IL-4 levels were similar in patients and controls, increased IL-10 levels were found in patients. Exogenous addition of IL-10 resulted in reduced MGC formation. Contrastingly, when IL-4 was added exogenously, it led to increased MGC formation. The effects of both IL-10 and IL-4 were reversed upon addition of their respective antibodies.

**Conclusion:**

The findings suggest that one of the factors contributing to the disease could be the effect of cytokines on the functionality of monocytes, which are crucial in the fight against the organism. Significantly reduced MGC formation was observed on addition of IL-10. The findings imply an overriding role of IL-10 in MGC formation. The suppressive effect of IL-10 on MGC formation was further confirmed by addition of IL-10 neutralizing antibody.

## Introduction

Tuberculosis is the second leading cause of mortality after human immunodeficiency virus (HIV) [1] Despite extensive research, there are several unanswered questions regarding the pathology of tuberculosis and the host response to overcome the disease. Granulomas are pathologic hallmarks of tuberculosis. The fate of the granuloma differs remarkably in an immunocompetent person in whom it undergoes calcification and eventually heals, as compared to an immunodeficient person where it leads to necrosis, cavitation and thereby spread of the disease [2]. Thus, on one hand, granuloma seems to serve as a shelter for harboring the bacteria but on the other hand, the T cell mediated activation in the granuloma results in bactericidal or bacterio-static effect on the tubercle bacilli [3]. Granulomas characteristically consist of multinucleated giant cells (MGC) formed from fusion of monocytes [4]. While MGCs are unable to mediate bacterial uptake, their NADH oxidase activity and antigen presentation properties are conserved [5]. MGC therefore seems to be dedicated to destruction of bacilli already ingested in the previous stages of differentiation.

 However, the factors and mechanisms involved in the formation of MGC are not clear. There are several cytokines which are key players in the immune response occurring within the granulomas. Several studies have indicated the importance of a balance between Th1 (T helper cell 1) cytokines and Th2 (T helper cell 2) cytokines in the pathology of tuberculosis [6-9] Interleukin-2 which is a Th1 cytokine facilitates T cell replication and promotes cellular immunity apart from being a crucial factor for granuloma formation [10]. Conversely, Th2 cytokine IL-10 inhibits T cell proliferation by down regulating the production of IL-2 [11]. IL-10 is also known to contribute significantly to formation of a disorganized granuloma [2,12]. Several studies have demonstrated the immunosuppressive role of IL-10 cytokine in human and animal models [13,14]. Experimental evidence also suggests that use of IL-10 specific neutralizing antibody resulted in enhancement of tuberculosis proliferation [15]. IL-4 is another Th2 cytokine that has been shown by various studies to be involved in MGC formation [16,17]. In the present study we describe, for the first time, the role of IL-10 in MGC formation, in the continued presence of IL-4.

Among other cytokines, TNF-α also has a critical role in the maintenance of the granuloma and the formation of reactive nitrogen intermediates (RNI) that are formed in the activated macrophages [9,18]. Using cytokine specific monoclonal antibodies against TNF-α, a 5-10 fold increase in reactivation of tuberculosis was observed [18]. TGF-β on the other hand has been found to oppose the action of TNF-α thus contributing to the pathology of the disease [19,20]. Hence, an in-depth study of these cytokines on in vitro MGC formation, which is a correlate of in vivo granuloma, would provide vital clues in understanding the pathology of the disease.

Several groups have attemped to study in vitro MGC formation using monocytes from healthy control individuals [4,5]; however, no study appears to have been made to ascertain whether monocytes from patients and controls behave differently in response to cytokines produced by (patient or control) mononuclear cells following Mtb specific stimulation in vitro. To test this hypothesis, monocytes from patients and controls were incubated with culture supernatant of Con A and PPD stimulated mononuclear cells of both these groups. Subsequently, MGC formation was observed microscopically and along with this the various cytokine levels in culture supernatant were also analyzed. It was observed that patient monocytes behaved differently in the presence of cytokine containing supernatants from patients in comparison to healthy controls. Further, when the cytokine levels in culture supernatants were analyzed, it was found that IL-10 was consistently high in culture supernatants of patients than controls. However, the levels of IL-4 were found to be similar in both the groups. To further investigate the relative role of IL-4 and IL-10 in MGC formation, these cytokines and their respective neutralizing antibody were exogenously used with the culture supernatant reiterating the immunosuppressive role of IL-10 in in vitro MGC formation, in the continued presence of IL-4.

## Materials and Methods

### Study population

All patients (from SSG hospital, Vadodara) included in the study were newly diagnosed tuberculosis patients with roentgenographic findings (chest X-ray) consistent with TB and sputum positive for AFB (acid fast bacilli) and were human immunodeficiency virus negative. Asymptomatic healthy controls were also included in this study. Six males and four females of 20 to 50 years of age were included in each group. Blood samples were collected in EDTA vaccutainer. All procedures used in the study were approved by Institutional Ethics Committee for Human Research of the Faculty of Science, M. S. University of Baroda. Written informed consent was obtained from all subjects.

### Isolation of peripheral blood mononuclear cells (PBMC)

PBMC were separated from 10 mL blood samples collected from each individual by density gradient centrifugation. Blood was first layered on equal volume of Histopaque-1077 (SIGMA Chemicals, USA) and centrifuged at 400 x g for 20 mins at room temperature. The buffy coat at the interphase was carefully collected and the mononuclear cells were washed twice with phosphate-buffered saline (PBS) before being resuspended in RPMI-1640 medium containing 10% fetal calf serum.

### *In vitro* MGC formation

10^6^ PBMCs were seeded onto four well LabTek chamber slides (Nunc). Following two hours at 37°C and 5% CO2 the unadhered population was removed by repeated vigorous washing with RPMI 1640 culture medium. Subsequently, 2 X 10^5^ cells of the above collected unadhered population of patients as well as healthy controls were seeded in 24 well plates respectively. Over 90% of the adherent cells were identified as monocytes by morphological criteria and ﬂuorescence-activated cell sorting (FACS) analysis for the presence of CD14. Trypan blue dye exclusion was used to determine viability. The unadhered peripheral mononuclear cells were used as the source of lymphocytes and were stimulated for 3 days with 16µg/ml Concanavalin A (Con A) or 10µg/ml purified protein derivative (PPD, a kind gift from Kris Huygen, O.D. Communicable and Infectious Diseases, Brussels). The unstimulated cells of patients and healthy controls were taken as negative control. The supernatant from each of these wells were collected and then added at 50% final concentration to various monocyte cultures. The supernatant obtained from healthy control unstimulated cells (C Med-SN) and those stimulated with Con A (C Con A-SN) and PPD (C PPD-SN) were then incubated with autologous healthy control as well as patient monocytes respectively. Also the supernatant obtained from patient unstimulated cells (Pt Med-SN) and those stimulated with both Con A (Pt Con A -SN) and PPD (Pt PPD-SN) were incubated with patient monocytes. Following incubation for 3 days, the monocytes were stained using Leishman stain and microscopic examination was carried out to study the size and fusion rate of MGC formation. The fusion rate was calculated as the number of nuclei within MGC (more than two nuclei per cell) in a given area per total number of nuclei in that same area: fusion rate (%) = (number of nuclei within MGC/total number of nuclei counted) X 100 [4]. From each preparation 100-150 cells were selected from representative fields for calculation of fusion rate.

### Cytokine ELISA

Cell culture supernatants were stored at -20°C until analyzed. The levels of cytokines IL-2, IL-10, TNF-α and TGF-β was analyzed using sandwich ELISA immunoassay kits from GE Healthcare as per the manufacturer’s instructions. IL-4 was analysed using commercially available ELISA kit (PeproTech EC Ltd,. London, UK).The absorbance was measured at 450 nm (IL-2, IL-10, TNF-α, TGF-β) and 405nm (IL-4) respectively and compared with the respective standard curve of the cytokines.

### Effect of IL-10 and anti-IL-10 on MGC formation

For determining the role of IL-10 in MGC formation, IL-10 (50 ng/mL) (Peprotech, USA) as well as anti-IL-10 (5 μg/mL) (α-IL-10, Peprotech, USA) neutralizing antibody was used. Blood samples from healthy controls were taken (n=5) and in vitro MGC formation was observed as described above in 8 well Lab-tek chamber slide. The monocytes were incubated with autologous control culture supernatant having autologous Med-SN, Med-SN + IL-10, ConA-SN, ConA-SN + IL-10, ConA-SN + αIL-10 and ConA-SN + IL-10 + αIL-10. The fusion rate was observed following three days of incubation and staining the slides with Leishman stain. 

### Effect of IL-4 and anti-IL-4 on MGC formation

The effect of IL-4 was also studied in the same manner, using IL-4 (20ng/ml) and anti-IL4 (5 μg/mL). The monocytes obtained from five healthy controls were incubated with autologous control culture supernatant having autologous Med-SN, Med-SN + IL-4, Med-SN + IL-4 + αIL-4, ConA-SN, ConA-SN + IL-4, ConA-SN + αIL-4 and ConA-SN + IL-4 + αIL-4. The fusion rate was observed following three days of incubation and staining the slides with Leishman stain.

### Statistical analysis

Statistical analysis was performed using GraphPad Prism software (GraphPad, San Diego, CA, USA). The Mann–Whitney U-test was applied for group differences. A Bonferroni–Holm procedure was used to correct for multiple comparisons between groups.

## Results

**Figure 1:**
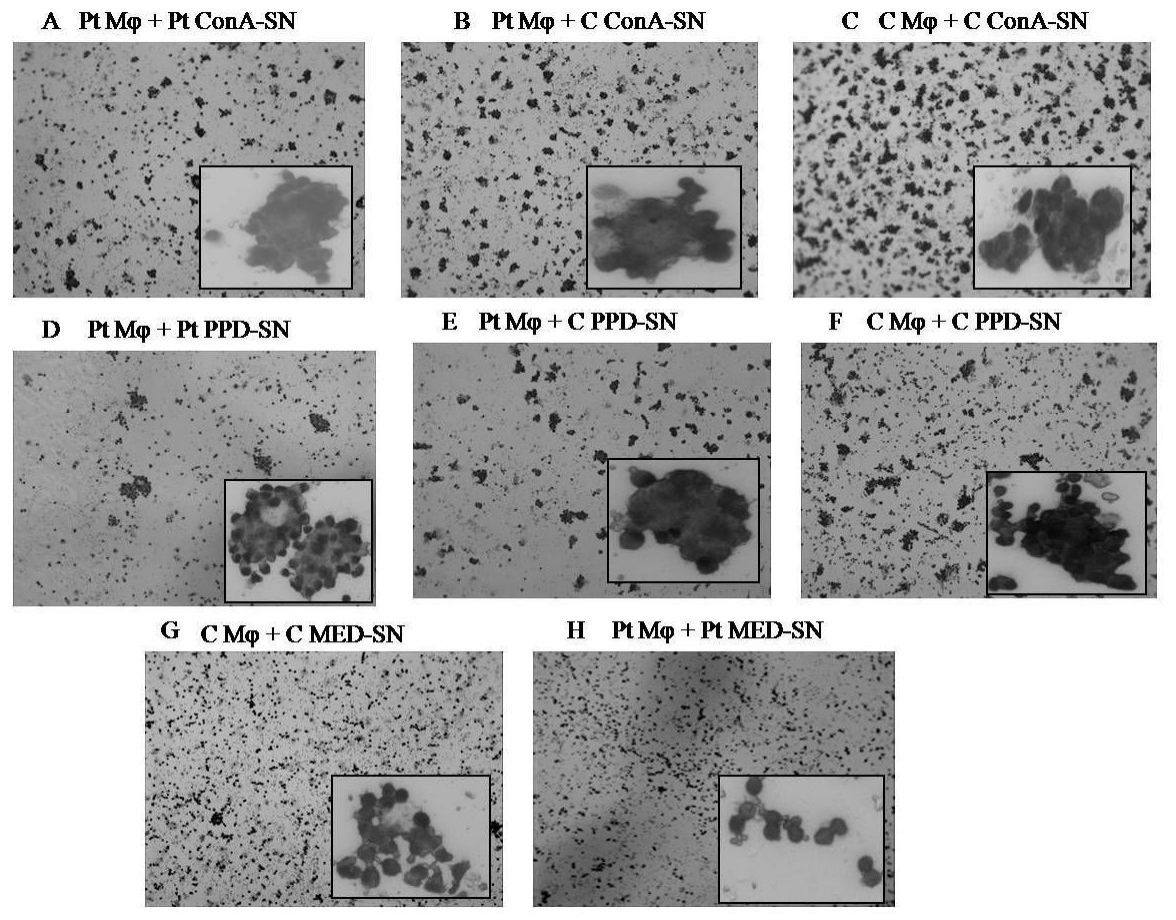
Leishman stained representative pictures of in vitro MGC. Monocytes from patients (Pt) and controls (C) were incubated with culture supernatant derived from unstimulated control (Med-SN), ConA stimulated (ConA-SN) and PPD (PPD-SN) stimulated peripheral mononuclear cells obtained from patients and controls respectively. Patient monocytes were incubated with Pt ConA-SN (a), C ConA-SN (b), Pt PPD-SN (d), C PPD-SN (e) and Pt Med-SN (h). Control monocytes were incubated with C ConA-SN (c), C PPD-SN (f) and C Med-SN (g).

**Figure 2:**
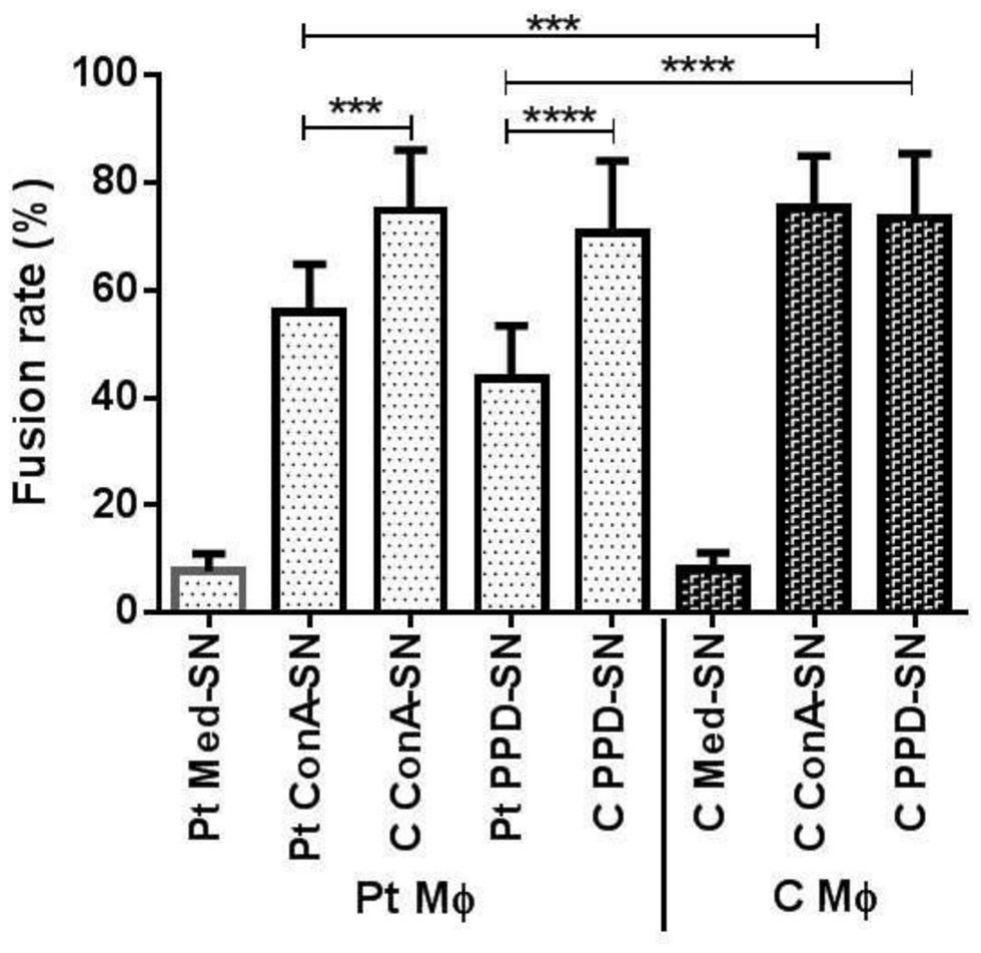
Graph showing percent fusion rate of in vitro MGC formation. Monocytes from patients (Pt Mφ) (n=10) and controls (C Mφ) (n=10) were incubated with supernatant derived from unstimulated control (Med-SN), ConA stimulated (ConA-SN) and PPD stimulated (PPD-SN) peripheral mononuclear cells obtained from patients and controls respectively. Comparability of groups was analyzed by Mann–Whitney U-test. A Bonferroni–Holm procedure was used to correct for multiple comparisons between groups. *p < 0.05, **p < 0.01, ***p < 0.001, ****p<0.0001.

In the present study we have examined and compared the ability of patient and control monocytes to form MGC by treating them with cytokine containing supernatant of ConA or PPD treated nonadherent mononuclear cells. Figure 1 shows representative pictures of in vitro MGC formation by patient and control monocytes treated with various supernatants. The percent fusion rate was calculated as the ratio of the number of nuclei forming MGC to the number of total nuclei counted as given in Figure 2. As shown in Figure 1, the monocytes of patients (Figure 1g) and controls (Figure 1h) incubated with their respective unstimulated supernatant of nonadherent mononuclear cells were found to be dispersed and very low fusion was observed (Control- 7.8%, Patient- 8.1%). However, the fusion rate of patient monocytes incubated with C ConA-SN (74.8%) (Figure 1b) was significantly higher (p=0.0003) than that with Pt ConA-SN (55.9%). (Figure 1a). Similarly, the fusion rate was significantly high (p<0.0001) in patient monocytes incubated with C PPD-SN (70.6%) (Figure 1e) than Pt PPD-SN (43.5 %) (Figure 1d). 

It can also be seen from Figure 1 that the control monocytes incubated with C ConA-SN (75.2%) and C PPD-SN (73.4%) had significantly higher (p=0.0002, p< 0.0001) fusion rates than patient monocytes incubated similarly with autologous supernatants respectively. However no significant difference was observed with patient or control monocytes when incubated with ConA-SN or PPD-SN derived from control peripheral mononuclear cells (Figure 1b, 1c, 1e, 1f).

**Figure 3:**
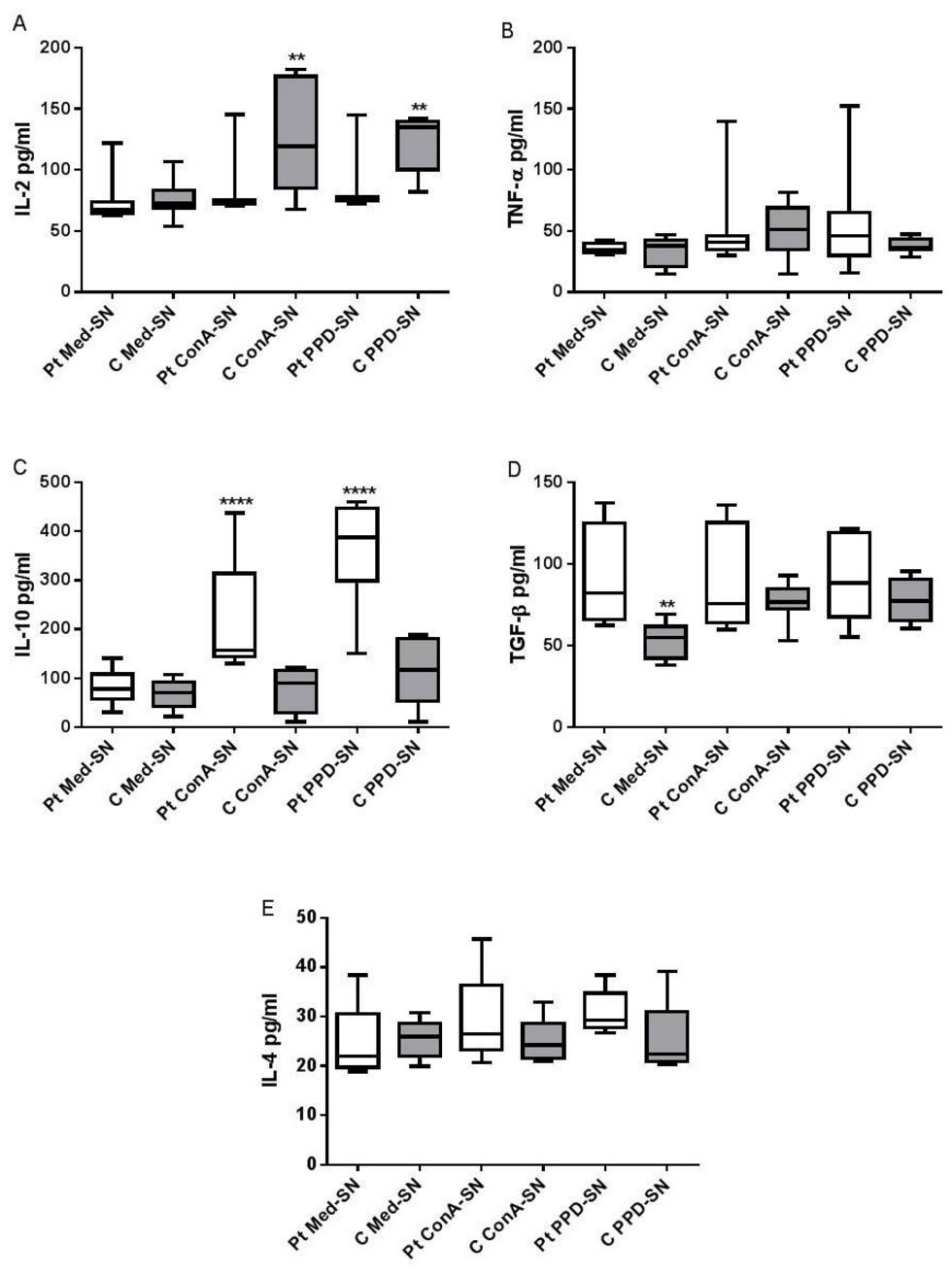
Cytokine analysis of culture supernatants: Culture supernatants obtained from peripheral mononuclear cells of patients (Pt) and controls (C) incubated with ConA (ConA-SN) and PPD (PPD-SN). Control wells were seeded with cells without any antigen/mitogen (Med-SN). The levels of (A) IL-2 , (B) TNF-α, (C) IL-10 (D) TGF-β and (E) IL-4 were analysed by ELISA. Comparability of groups was analyzed by Mann–Whitney U-test. A Bonferroni–Holm procedure was used to correct for multiple comparisons between groups. *p < 0.05, **p < 0.01, ***p < 0.001, ****p<0.0001.

We analyzed the levels of IL-2, TNF-α, IL-4, IL-10 and TGF-β1 in patient and control supernatant by ELISA (Figure 3). IL-2 was significantly higher in C ConA-SN (p=0.0016) and C PPD-SN (p=0.0025) than Pt ConA-SN and Pt PPD-SN respectively (Figure 3a). Another Th1 cytokine, TNF-α however was not found to be significantly different in ConA-SN or PPD-SN of patients and controls (Figure 3b). Also, neither IL-2 nor TNF-α, showed any difference in the spontaneous release without any antigen stimulation in both the groups.

IL-10, on the other hand showed significantly high levels in Pt ConA-SN (p<0.0001) and Pt PPD-SN (p<0.0001) compared to C ConA-SN and C PPD-SN respectively (Figure 3c). On the contrary the other Th2 cytokine TGF-β did not show any significant difference when Pt ConA-SN and Pt PPD-SN were compared to C ConA-SN and C PPD-SN respectively (Figure 3d). Although IL-10 levels in Med-SN between patients and controls did not show any significant difference, TGF-β1 levels were found to be significantly high (p = 0.001) in patients.

**Figure 4:**
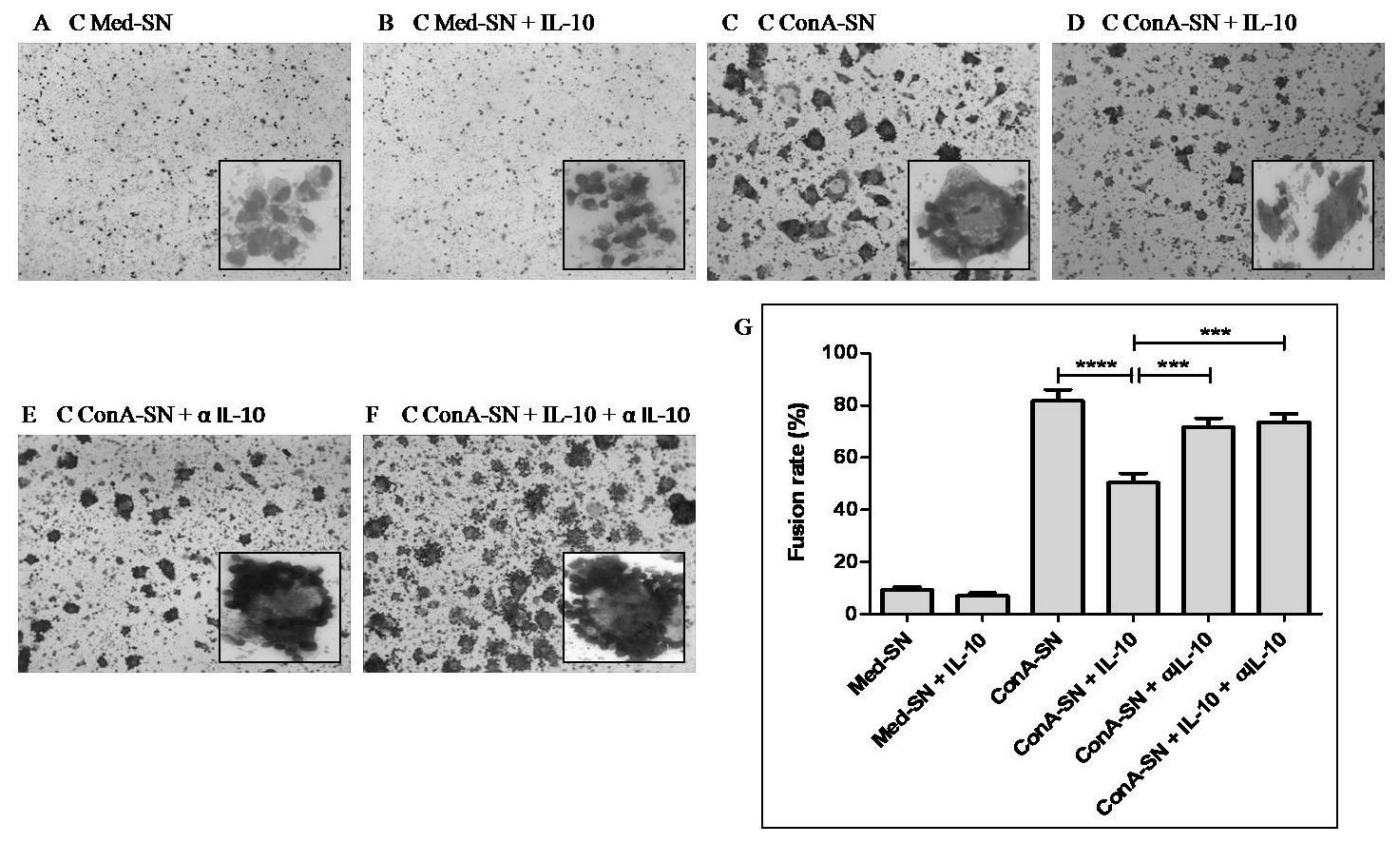
Effect of IL-10 and anti-IL-10 antibody (α IL-10) on in vitro MGC formation. Figure shows representative Leishman stained pictures of MGC formation and fusion rate of monocytes obtained from healthy controls (n=5) incubated with autologous culture supernatants. (A) unstimulated culture supernatant (Med-SN) (B) Med-SN + IL-10 (C) ConA stimulated culture supernatant (ConA-SN) (D) ConA-SN + IL-10 (E) ConA-SN + α IL-10 (F) ConA-SN + IL-10 + α IL-10 (G) Graph showing effect of IL-10 and anti-IL-10 on percent fusion rate of in vitro MGC formation. Comparability of groups was analyzed by Mann–Whitney U-test. A Bonferroni–Holm procedure was used to correct for multiple comparisons between groups. *p < 0.05, **p < 0.01, ***p < 0.001, ****p<0.0001.

In order to confirm the role of IL-10 in MGC formation, control monocytes were incubated independently with autologous Med-SN, Med-SN +IL-10, ConA-SN, ConA-SN + IL-10, ConA-SN + αIL-10 and ConA-SN + IL-10 + α IL-10 respectively. Figure 4 shows the fusion rates for these treatments. No significant difference in the fusion rate was observed when control monocytes were incubated with Med-SN (9.2 %) or Med-SN + IL-10 (7%). However, the fusion rates decreased significantly (p<0.0001) following treatment of monocytes with ConA-SN + IL-10 (50.4%) as compared to ConA-SN (81.8%). Although aggregates were observed in the former, the rate of fusion was significantly reduced compared to the latter. To further confirm the effect of IL-10, when IL-10 neutralizing antibody was used, it was found that the rate of fusion in the presence of ConA-SN containing both IL-10 and neutralizing antibody (73.6%) was significantly increased (p=0.0004) compared to ConA-SN+ IL-10 alone (50.4%). There was no significant effect observed when anti-IL-10 was included solely with ConA-SN (71.6%).

**Figure 5:**
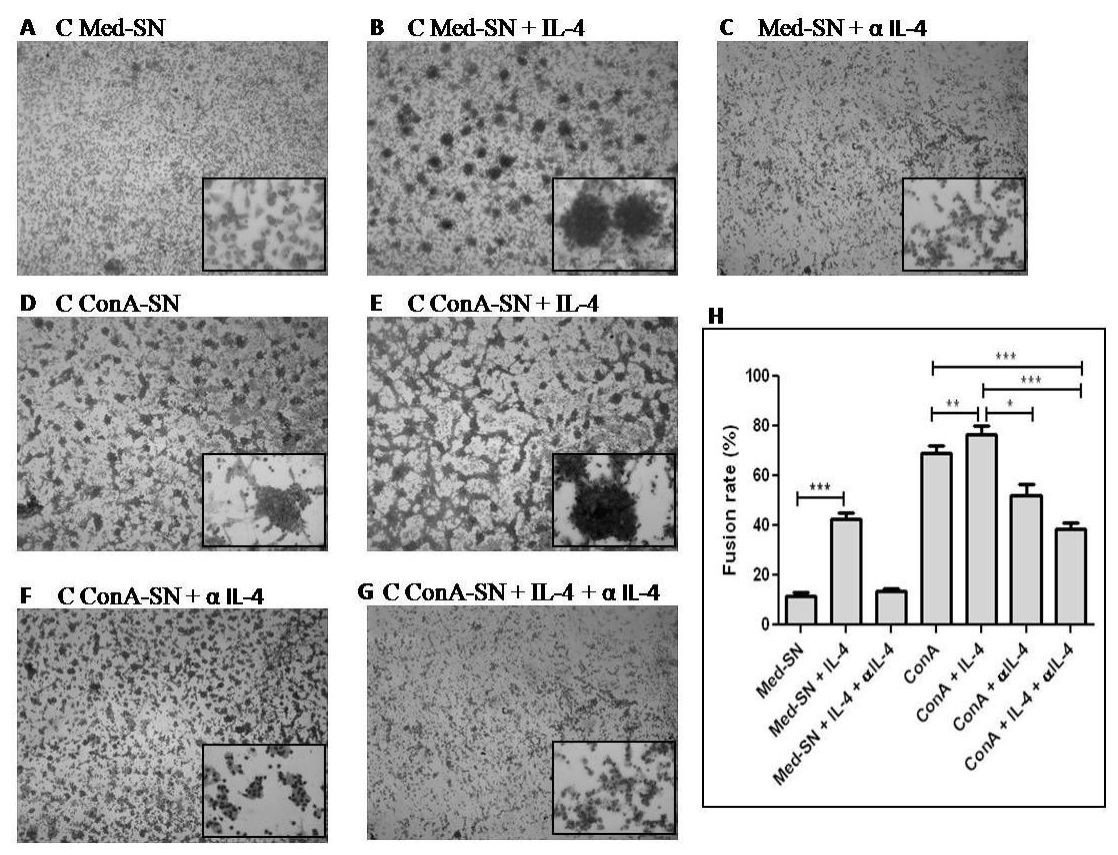
Effect of IL-4 and anti-IL-4 antibody (α IL-4) on in vitro MGC formation. Figure shows representative Leishman stained pictures of MGC formation and fusion rate of monocytes obtained from healthy controls (n=5) incubated with autologous culture supernatants. (A) unstimulated culture supernatant (Med-SN) (B) Med-SN + IL-4 (C) Med-SN + IL-4 + αIL-4 (D) ConA stimulated culture supernatant (ConA-SN) (E) ConA-SN + IL-4 (F) ConA-SN + α IL-4 (G) ConA-SN + IL-4 + α IL-4 (H) Graph showing effect of IL-4 and anti-IL-4 on percent fusion rate of in vitro MGC formation. Comparability of groups was analyzed by Mann–Whitney U-test. A Bonferroni–Holm procedure was used to correct for multiple comparisons between groups. *p < 0.05, **p < 0.01, ***p < 0.001, ****p<0.0001.

In contrast to IL-10, the levels of IL-4 were not found to be different in the culture supernatant of patients and controls whether or not they were stimulated by ConA or PPD (Figure 3). However, when IL-4 was added exogenously along with autologous Med-SN and ConA-SN to evaluate in vitro MGC formation, it was observed that the fusion rate was significantly increased in both the cases (p= 0.0001 and p= 0.0037 respectively) (Figure 5). Furthermore, upon addition of IL-4 neutralizing antibody, this effect was reversed and significant decrease in the fusion rate was observed in both Med-SN (p= 0.0001) as well as Con-SN (p= 0.0003) (Figure 5).

## Discussion

We report here the comparison in MGC formation by monocytes of tuberculosis patients and healthy controls using an in vitro model. Several groups have used in vitro granuloma model in the past to gain better understanding of immunopathology of tuberculosis using monocytes of only healthy controls [4,5,21]. Gasser and Most [4] reported the in vitro differentiation of monocytes to MGC using mycobacteria in combination with BCG stimulated T cell supernatant of control lymphocytes. In another study, Lay et al. studied an in vitro granuloma model and reported that MGC formed in Mtb induced granuloma loose their ability of bacterial uptake but retain their bactericidal activity following MGC formation [5]. However, no study appears to have been done to compare the monocytes of tuberculosis patients and healthy controls in their in vitro MGC forming ability with particular reference to the relative roles of IL-4 and IL-10. In the present study, we have examined the differences in MGC formation of patient and control monocytes (adhered cells) when incubated with their respective culture supernatants obtained following ConA or PPD stimulation of autologous peripheral mononuclear cells (unadhered cells). MGC formation was also evaluated for patient monocytes incubated with ConA or PPD stimulated control peripheral mononuclear cell (unadhered cells) culture supernatant. We have observed that the rate of fusion and MGC formation in case of patient monocytes was much less than that of healthy controls when incubated with their autologous supernatant (Figure 1a,1d,1c,1f). Furthermore, when the same patient monocytes were incubated with ConA or PPD stimulated control peripheral mononuclear cell culture supernatant the rate of fusion was surprisingly increased (Figure 1b,1e). These results reflect that the patient monocytes have the ability to form MGC. However, the difference in the results obtained when patient monocytes are treated with patient supernatant or control supernatant reflects upon the difference in the cytokine produced by the two groups. 

Organized granuloma is a prerequisite to limit mycobacterial infection, the fate of which is decided by both Th1 as well as Th2 cytokines. Hence we investigated the IL-2, TNF-α, IL-4, IL-10 and TGF-β levels present in the respective lymphocyte supernatants of patient and healthy controls by ELISA. IL-2, which is a Th1 cytokine, contributes to cellular immunity by facilitating T cell replication and granuloma formation [10]. IL-2 levels were found to be significantly increased in PPD stimulated lymphocytes of controls but not patients (Figure 3). This is in agreement with Birkness et al. who observed MGC formation in control PBMCs and macrophages following treatment with IL-2, although not including patient lymphocytes in their study [21]. However, contrary to the above two observations, Gasser and Most [4] conclude from their study that there was no correlation between IL-2 levels and in vitro MGC formation. Therefore, it appears that in accordance with the observation of Birkness et al. [21], in the present study, IL-2 which is produced in response to PPD may contribute to the higher fusion rate seen with control PPD-SN compared to patient PPD-SN.

The role of TNF-α is quiet varied as demonstrated by many studies that have indicated that TNF-α did not have a role in MGC formation [22,23]. Studies have also highlighted a role for TNF-α in MGC formation and maintenance of granuloma [21]. In the present study, we observed variable levels of the cytokine following stimulation with ConA or PPD in case of both patients and control cells (Figure 3). This indicates that TNF-α may not be absolutely essential for MGC formation while the presence of it does not interfere with MGC formation. 

IL-4 is a Th2 cytokine which increases TNF-α toxicity thereby aggravating tissue damage and inducing fibrosis of granuloma leading to enhanced immunopathology [24]. Several studies have acknowledged and demonstrated the role of IL-4 in MGC formation [16,17]. In addition, excessive IL-4 production has also been correlated with active TB and a depressed Th1 response [25]. However, as shown in Figure 3, the levels of IL-4 was found to be similar in both patients and controls whether or not they were stimulated with ConA or PPD. 

IL-10 is a potent anti-inflammatory cytokine that deactivates macrophages, down regulates Th1 response and limits antigen presentation. High levels of IL-10 have been associated with disorganized granuloma formation [2]. We observed significantly high levels of IL-10 in patient PPD-SN and patient ConA-SN but not in case of control supernatants (Figure 3). This implies that patients have higher population of T cells that can produce high levels of IL-10 compared to that of control lymphocytes. Similar observations were made by Pereira et al. [26] where in case of both ex vivo and in vivo exposure of monocytes to Mtb, the IL-10 and TNF-α levels were significantly high in active tuberculosis patients compared to healthy controls. 

TGF-β, an anti-inflammatory cytokine has also been associated with disorganized granuloma formation [2]. In the present study, we did not observe any significant difference in the levels of TGF-β1 following stimulation of patient and control mononuclear cells with ConA or PPD in comparison to their respective unstimulated culture supernatants. Interestingly, TGF-β1 levels observed in unstimulated culture supernatants was significantly high in patients as compared to controls (Figure 3). Furthermore, this heightened level of TGF-β1 was not increased any further following stimulation with either ConA or PPD. 

Studies have also shown elevated levels of TGF-β [27] following stimulation of PBMC isolated from tuberculosis patient with PPD. The contrasting results obtained for TGF-β in the present study may be attributed to the population of cells used. While in the studies mentioned above PBMCs were used, in the present study we used unadhered cells as a source of lymphocytes. These cells are devoid of monocytes and as evident from the literature monocytes are the principle source of TGF-β [28]. Also among the lymphocytes NK cells [28] and Tregs [29] are known to produce TGF-β. However these cells constitute very small population of lymphocytes. This may explain the insignificant change in the levels of TGF-β in stimulated cells in contrast to IL-10 which was found to be significantly high. IL-10 is produced by B lymphocytes, dendritic cells, Th2 cells and Tregs.

Interestingly, Th2 cytokines IL-10 and IL-4 have opposite effects on MGC formation. On one hand IL-10 has been correlated with disorganized granuloma formation whereas IL-4 is known to favour MGC formation. In order to investingate which of the two Th2 cytokines has a predominant effect on MGC formation, monocytes were treated with exogenous cytokines in the presence and absence of their corresponding neutralizing antibodies. The results confirm the immune suppressive role of IL-10 wherein inclusion of IL-10 led to reduced MGC formation which was reversed by addition of anti-IL-10 antibody. Conversely, addition of IL-4 led to significant increase in MGC formation and the effect was neutralized following addition of anti-IL-4 antibody. These results therefore demonstrate that IL-10 appears to override the effect of IL-4 in patient supernatants which in turn is responsible for reduced MGC formation.

As evident from Figure 3, control culture supernatant stimulated with ConA as well as PPD, contained high levels of IL-2. Despite the presence of IL-2, since IL-10 cytokine was effective in reducing the fusion rates of control monocytes we did not feel the need to neutralize IL-2 to observe the effect. 

This study demonstrates that patient and control monocyte have similar potential in MGC formation in vitro as evident from the fusion rates given in Figure 2. However, the cytokine producing ability of their lymphocytes differs with regard to both Th1 cytokine IL-2 and Th2 cytokines IL-4, IL-10 and TGF-β. The role of IL-4 in MGC formation was confirmed in the present study. In addition, we have also confirmed the immunosuppressive role of IL-10 in MGC formation in patients as compared to controls, despite similar levels of IL-4 found in both the groups. Hence, this study establishes the unimpaired potential of patient monocytes in MGC formation and that the apparent impaired functionality of patient monocytes in MGC formation is likely due to the presence of IL-10. 
